# Taylor Series Interpolation-Based Direct Digital Frequency Synthesizer with High Memory Compression Ratio

**DOI:** 10.3390/s25082403

**Published:** 2025-04-10

**Authors:** Kalle I. Palomäki, Jari Nurmi

**Affiliations:** Wireless Research Center, Tampere University, 33720 Tampere, Finland; jari.nurmi@tuni.fi

**Keywords:** direct digital frequency synthesis, Taylor series interpolation, FPGA, memory compression ratio, spurious free dynamic range

## Abstract

A common challenge in direct digital frequency synthesizers (DDFSs) is obtaining high memory compression while maintaining good output signal purity. To address this challenge, in this paper, we present a 16-bit, quadrature direct digital frequency synthesizer (DDFS) that utilizes the second-order Taylor series polynomial interpolation in the phase-to-amplitude conversion. In this approach, the sinusoidal signal is divided into multiple segments, and for each segment, related values are stored into a look-up table (LUT). The amplitude values for each segment are calculated using the stored LUT values and the second-order Taylor series polynomial interpolation. A Python-based model was created to optimize the number of segments, and the resulting design was coded using register-transfer level VHDL. The design is synthesized and implemented on an AMD Artix 7 FPGA, and the implementation results are presented. We show that the proposed design is capable of reaching a very high memory compression ratio of 5178:1. Additionally, the design generates both sine and cosine with high spectral purity utilizing a low number of FPGA resources compared to previous work. With 107 logic slices and 3 DSP slices, the design reaches a spurious-free dynamic range (SFDR) of −102.9 dBc.

## 1. Introduction

A direct digital frequency synthesizer (DDFS) is used to generate digital signals with various kinds of waveforms, for example sawtooth, triangle, or sinusoidal signals. In this paper, we are focusing on digital sinusoidal signal generation, since sinusoidal signals have broad application areas across multiple industries. The original idea for the DDFS was introduced in 1971 by Tierney, Rader, and Gold [1].

Nowadays, a DDFS has become a very common digital block used in many designs. For example, in telecommunications, a DDFS is used broadly in wireless communications and software radio [2,3]. Also, in more modern networks, such as 5G, the DDFS has been a common research topic [4,5,6]. The improvements in DDFS technologies have enabled its use in several different applications in many diverse fields such as medical instruments, radars, and even with nuclear science in neutrino observatories [7,8,9,10]. Furthermore, the recent advancements in DDFS have enabled it to be used in several quantum technology fields, such as quantum communications, quantum computing, and quantum sensing. In quantum communications, the DDFS is used as it provides a versatile method for generating nearly any arbitrary combination of phase and frequency [11]. In modern-day quantum computers utilizing trapped-ion or spin qubits, the DDFS is used in the control electronics [12,13,14], and in quantum sensing, the DDFS has been applied for example in cold atom gravimeters [15]. These new emerging application fields have given rise to the development of new hardware designs to enable more efficient DDFS implementation. We will start by introducing the generic DDFS architecture.

**Figure 1:**
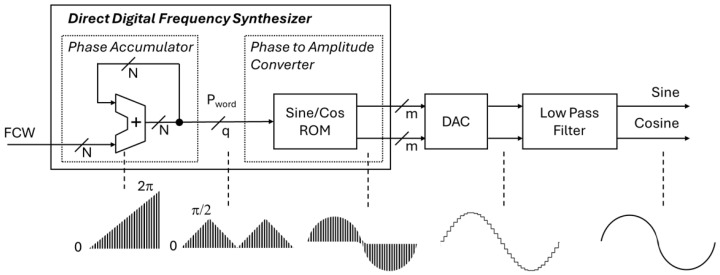
Generic architecture of a LUT-based direct digital frequency synthesizer.

The original principle for the DDFS design used a read-only memory (ROM) for storing the amplitude values and a modulo-2 counter output as the ROM address [1]. The generic architecture for the ROM-based approach is given in Figure 1. The typical DDFS implementation has four main components. In the digital domain, the first component is the phase accumulator (PA), which contains a phase register and an adder. At each clock cycle, the adder sums the frequency control word (FCW) to the phase register value. Each phase register overflow represents a full 2π period of the sinusoidal signal, and the FCW is used to set the frequency of the output signal, $f_{out}$

(1) $$f_{out}=\frac{FCW}{2^{N}}\cdot f_{clk},$$

where $f_{clk}$ is the system clock frequency. Furthermore, the bit width of the FCW, *N*, defines the frequency resolution, Δf, which is given below in Equation (2)

(2) $$\Delta f=\frac{f_{clk}}{2^{N}}.$$

From the frequency resolution perspective, it is preferable to have large *N* width, and typically the FCW is rather long, from 32 to 48 bits. Based on (2), for example with a 100 MHz system clock, the frequency resolution provided by the 32-bit FCW is 0.023 Hz and by the 48-bit FCW is $3.55\cdot 10^{-7}$ Hz.

The PA output is a phase word, $P_{word}$, for the second digital component, the phase-to-amplitude converter (PAC). The PAC can be implemented using different approaches, but most commonly, some kind of memory is involved in the design. The generic architecture uses the ROM to store the sine and cosine amplitude values, and $P_{word}$ is used as the ROM address. Before the PA output can be used, the PA output needs to be truncated to *q* bits in order to keep the resulting ROM size manageable. However, this truncation reduces the accuracy, resulting in inferior output signal quality and spurs in the spectrum. In DDFS designs, it is important to reduce these spurs, as output signal quality is one key metric of the performance of the DDFS. The signal quality is reported using the spurious free dynamic range (SFDR), which is the ratio of the fundamental signal to the strongest spurious signal. A commonly known equation in DDFS designs for estimating the relationship between the $P_{word}$ bit width, *q*, and the SFDR is given below in Equation (3) [16]

(3) SFDR(dBc)≈6.02q−3.92dB.

This relationship implies that a large *q* value is preferred from the signal quality perspective. However, each bit in $P_{word}$ width doubles the required memory, leading to increased design area and more expensive hardware implementation. Therefore, over the past two decades, a lot of research has been put into optimizing the size of the ROM [17,18,19,20,21]. A common metric to evaluate the optimization efficacy is to compare the compressed memory size to that of the equivalent generic DDFS implementation with minimally compressed or completely uncompressed memory. The uncompressed memory size is calculated by $2^{q}\cdot m$, where *m* is the amplitude bit width of the sinusoid. If both sine and cosine waves are generated, then the equivalent uncompressed memory size is $2^{q}\cdot 2m$. Memory compression schemes often reduce the accuracy of the amplitude representation and, therefore, some compression algorithms come at the expense of a reduced SFDR. Therefore, it is key to find a balance that maximizes the memory compression ratio and maintains a high SFDR.

Once the amplitude values are generated, they are converted into analog format using a digital-to-analog converter (DAC). The resulting signal is typically filtered using a low-pass filter to create a more pure output. In this paper, we are proposing a method for improving hardware implementation of the digital components: that is, the phase accumulator and the phase-to-amplitude converter.

## 2. ROM Compression Methods

In this section, we will provide a brief overview of some of the most commonly used ROM compression methods. The first compression method discussed is based on the sine and cosine signal symmetry, but there are also several other methods to further compress the memory after the symmetry-based compression has been applied. These include, for example, the sine phase difference algorithm, the use of trigonometric identities, fine and coarse ROM division, interpolation methods, and Taylor series optimization [22,23,24,25,26]. In the scope of this paper, we will introduce below in more detail the symmetry, interpolation, and Taylor series-based methods for memory optimization.

### 2.1. Sine and Cosine Symmetry

One of the most commonly applied methods for DDFS ROM compression is to utilize the symmetry properties of the sine and cosine functions [27]. The first symmetry property is called the quarter-wave (QW) symmetry. The QW symmetry implies that a full sine or cosine period of 2π can be generated by using only the amplitude values in $\left[0,\frac{\pi }{2}\right]$. As an example, the QW symmetry for sine is given in (4).

(4) $$\sin\left(x\right)=\left\{\begin{array}{cc} \sin(x), & \mathrm{when}\;x\in \left[0,\frac{\pi }{2}\right]\\ \sin(\pi -x), & \mathrm{when}\;x\in \left[\frac{\pi }{2},\pi \right]\\ -\sin(x-\pi ), & \mathrm{when}\;x\in \left[\pi ,\frac{3\pi }{2}\right]\\ -\sin(2\pi -x), & \mathrm{when}\;x\in \left[\frac{3\pi }{2},2\pi \right] \end{array}\right.$$

As can be noticed, the full sine period is generated by rotating and negating the first quarter values.

When the DDFS system has both sine and cosine available, then it can take advantage of the second symmetry property that exists between sine and cosine. This symmetry is sometimes referred to as the sine and cosine eighth-wave symmetry. This property for the sine signal is given below.

(5) $$\sin\left(x\right)=\left\{\begin{array}{cc} \sin(x), & x\in \left[0,\frac{\pi }{4}\right]\\ \cos(\frac{\pi }{2}-x), & x\in \left[\frac{\pi }{4},\frac{\pi }{2}\right] \end{array}\right.$$

Based on (5), we can see that the sine values between $\left[0,\frac{\pi }{2}\right]$ can be generated by using both sine and cosine values between $\left[0,\frac{\pi }{4}\right]$. From these values, the full period can then be created by applying the QW symmetry.

In the proposed design architecture, both sine and cosine are present, and we will utilize both the quarter wave and the eighth-wave symmetry properties.

### 2.2. Interpolation Methods

In the interpolation approach, a period of the sine wave is divided into several, even hundreds of different segments. Often, to take advantage of the QW compression, this period is chosen to be $\left[0,\frac{\pi }{2}\right]$. The sine value is then approximated in these segments using some type of an interpolation equation. Most commonly, either linear, parabolic, or polynomial equations are applied for the approximation [20,21,25,28]. One of the benefits of the interpolation method is that it has been shown to provide a very high memory optimization. Two of the highest reported ROM compression ratios of 1103:1 and 1792:1 have been achieved using the interpolation-based memory optimization [18,21]. Many implementations of the interpolation method provide only sine signals instead of the supporting quadrature output signal. This is important to note especially when the implementations and their hardware resource utilization are compared.

In our proposed architecture, we are applying Taylor series approximation-based interpolation for the memory compression. However, instead of a hundred segments, we are using only a few tens of segments. Furthermore, the proposed architecture generates simultaneously both sine and cosine values.

### 2.3. Taylor Series Based Memory Optimization

Earlier works on applying Taylor series approximation for DDFS design include using the approximation directly for optimizing the stored memory values and using linear interpolation to improve the accuracy of the output signal [26,29]. The direct memory compression method has shown a memory compression ratio of 64:1 with an output SFDR of −97.04 [27]. Furthermore, the previously reported interpolation approach reported a 315:1 memory compression ratio and −77 dBc SFDR [29].

In this paper, we are presenting a different way of applying Taylor series approximation for an ROM compression scheme than what has been reported in the previous works. The proposed approach stores in memory three different values for each segment and applies Taylor series approximation-based computation to interpolate the remaining amplitude values for sine and cosine. We show that the proposed design achieves a high memory compression ratio while maintaining good spurious performance.

## 3. Memory Efficient DDFS Design Using Taylor Series-Based Interpolation

In this section, we introduce a reference design that utilizes the Taylor series approximation based interpolation in the PAC computation. In the method, we use the phase value within $\left[0,\frac{\pi }{4}\right]$ and divide that period into multiple segments. For each segment, a few values derived from the Taylor series approximation are stored into a memory, and the remaining amplitude values are computed by interpolating the signal using the second-order Taylor series. Since the Taylor series approximation requires the phase to be given in radians, the DDFS design uses a modified phase accumulator.

### 3.1. Optimizing the Taylor Series Approximation for DDFS Implementation

Taylor series approximation is a widely used method for approximating any continuous function near a point on the function, $x_{0}$ [30]. In this paper, we refer to this point as the evaluation point. The *k*th-order Taylor series approximation for a function f(x) is given below in (6):

(6) $$f\left(x\right)=\sum_{i=1}^{k}\frac{f^{\left(i-1\right)}\left(x_{0}\right)}{(i-1)!}{\left(x-x_{0}\right)}^{i-1}.$$

One of the features of the Taylor series approximation is that it is equally accurate to both positive and negative directions on the *x*-axis from the evaluation point. Additionally, the further away we move from $x_{0}$, the less accurate the approximation becomes.

**Figure 2:**
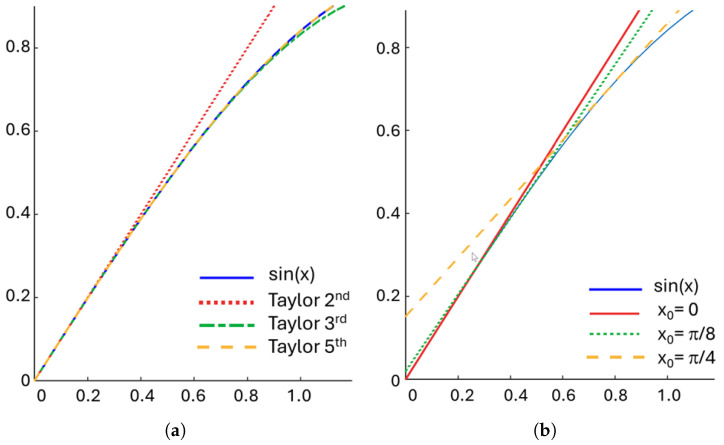
Taylor series approximation for sine between $\left[0,\frac{\pi }{4}\right]$. (**a**) Different order Taylor approximations with evaluation point at $x_{0}=0$ for sin(x), $x\in \left[0,\frac{\pi }{4}\right]$ (note: The fifth-order approximation overlaps sin(x) in this interval). (**b**) Increasing the accuracy of the second-order Taylor series approximation of sin(x) by using three evaluation points.

To improve the approximation accuracy of f(x), we can obviously increase the *k*, which adds more terms to the series. However, this also increases the computational complexity, as each new term introduces higher exponentiation than the previous term. This leads to several multipliers on the hardware implementation. Therefore, to keep implementation feasible, a low *k* value is preferred. A few optimization methods can be applied to enable the use of low *k* while maintaining sufficient approximation accuracy for the PAC implementation. The first method is to use the symmetry properties that were presented in the previous section. Since both sine and cosine are available, the symmetry around $\frac{\pi }{4}$ can be applied. Thus, only the amplitude values between $\left[0,\frac{\pi }{4}\right]$ need to be generated to create a full sine and cosine period. Looking at Figure 2a, it can be noted that the accuracy of the Taylor series approximation decreases the further the value is approximated from the evaluation point $x_{0}=0$. For example, the average error of the third-order Taylor approximation on sine between $\left[0,\frac{\pi }{4}\right]$ is 0.01296. This is over 20 times larger than the error in the same interval for the fifth-order Taylor approximation, which is $5.8649\cdot 10^{-4}$. Depending on the application, the fifth-order Taylor approximation accuracy for PAC implementation may be sufficient. On the hardware side, however, the fifth-order Taylor series approximation requires rather heavy computation, including five multipliers.

The second method to further reduce the required *k* is to use multiple evaluation points between $\left[0,\frac{\pi }{4}\right]$. An example of this principle is shown in Figure 2b, where three second-order Taylor series approximations, each having different evaluation points, are used to approximate sin(x). It can be seen that by changing the evaluation point as the phase increases, we can improve the accuracy of the second or Taylor series approximation. The use of multiple evaluation points reduces the error and enables the use of low *k*. On hardware implementation, the second-order Taylor approximation requires only one multiplier, which results into significantly lower complexity implementation compared with the fifth-order approximation.

### 3.2. Phase Accumulator Design

In the traditional DDFS, the PA adds the frequency control word to the accumulator value at every clock cycle and the truncated result, $P_{word}$, can be directly used as an address for ROM in the PAC. However, in the Taylor series approximation, the phase is part of the approximation equations and, therefore, the truncated phase has to be delivered to the PAC module in radians. This conversion to radians can be achieved by multiplying the truncated phase with $\frac{\pi }{4}$. Thus, the PA logic requires an additional fixed coefficient multiplier to perform the conversion. Since only one input of this multiplier changes, the logic implementation of the multiplier can be optimized. As an example, an 18-bit representation of π has half of the bits as 0. This means that this fixed coefficient multiplier generates 50% less partial products than a full 18-bit multiplier. This leads to both more efficient implementation and lower switching activity, which in turn reduces the power consumption of the multiplier.

One of the advantages of the Taylor series-based PAC design is that the $P_{word}$ width has a linear effect on the complexity of the PAC. In the traditional DDFS design, where the phase is used as an address for the ROM, each bit in the $P_{word}$ width doubles the size of the address space and thus the size of the ROM. In the traditional DDFS, this leads to exponential growth of the PAC logic. However, in the proposed design, the $P_{word}$ is not used as the memory address. Instead, the memory size is determined by the number of segments used. The $P_{word}$ width affects directly only the $x-x_{0}$ term in Equation (6), leading to a linear growth of hardware logic. Therefore, in the proposed method, we can use large a $P_{word}$ width. This is beneficial, since as was defined in (3), the $P_{word}$ width impacts directly the maximum attainable SFDR of the DDFS design.

**Figure 3:**
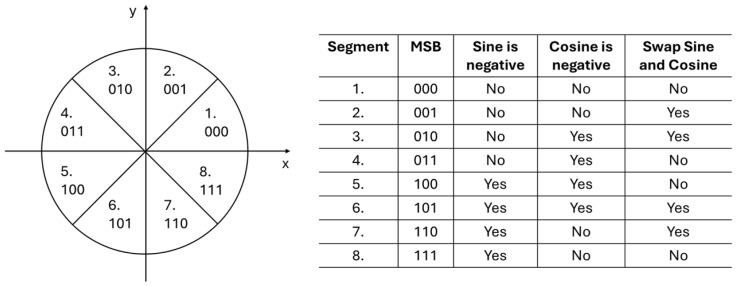
MSB control signals and their relation on the correct segment. Based on the segment, the sine and cosine can be negated or swapped with each other.

In order to take advantage of the sine and cosine symmetry over $\frac{\pi }{4}$, the phase has to be inverted after every $\frac{\pi }{4}$ segment. The inversion is simple to detect, as it is given by the third most significant bit (MSB) of the phase. To further enable the eighth-wave symmetry optimization, the top three MSBs are needed as control signals. They define in which of the eight segments the amplitude values need to be computed. The MSBs provide the correct sign for both sine and cosine and define when the sine value is used for cosine and vice versa. The functionality and how the three MSBs are used to define the correct position on the unit circle is shown in Figure 3.

The PA provides these MSBs directly to the symmetry logic, and the other multiplicand in the radian conversion is the phase starting from the fourth MSB. The PA in the reference design delivers to the PAC an 18-bit, triangle-formatted phase in radians. The operation of the PA is described using generic pseudocode in Algorithm 1.
**Algorithm 1** Modified phase accumulator operation using pseudocode.
    PHASE ACCUMULATOR    **Input:** Frequency control word (FCW), 32-bit    **Output:** Phase word, $P_{\mathrm{word}}$, 18-bit             whilesystemondo              ${\mathrm{Phase}}_{\mathrm{register}}=\left({\mathrm{Phase}}_{\mathrm{register}}+\mathrm{FCW}\right)\;\mathrm{mod}\;2$              ${\mathrm{Phase}}_{\mathrm{internal}}={\mathrm{Phase}}_{\mathrm{register}}\;\mathrm{mod}\;\frac{\pi }{2}\;//\;convert\;phase\;to\;\left[0,\frac{\pi }{2}\right]$              $\mathrm{if}\;{\mathrm{Phase}}_{\mathrm{internal}}>\frac{\pi }{4}\;\mathrm{then}\;\;\;//\;generate\;triangle\;formatted\;phase$                    ${\mathrm{Phase}}_{\mathrm{temp}}=\frac{\pi }{2}-\left({\mathrm{Phase}}_{\mathrm{internal}}\right)$              else                    ${\mathrm{Phase}}_{\mathrm{temp}}=\left({\mathrm{Phase}}_{\mathrm{internal}}\right)$              endif              $P_{\mathrm{word}}=\mathrm{truncate}\_\mathrm{to}\_P_{\mathrm{word}}\_\mathrm{bits}\left({\mathrm{Phase}}_{\mathrm{temp}}\right)$         endwhile


### 3.3. Phase to Amplitude Converter Design

As was presented in the previous section, dividing the approximation interval into multiple segments enables the use of lower-order Taylor series approximation equations. For the reference design PAC implementation, we will use the second-order equations. The second-order Taylor series approximation with respect to the evaluation point, $x_{0}$, is given below in (7) for sine

(7) $$sin\left(x\right)=sin\left(x_{0}\right)+cos\left(x_{0}\right)\left(x-x_{0}\right),$$

and in (8) for cosine

(8) $$cos\left(x\right)=cos\left(x_{0}\right)-sin\left(x_{0}\right)\left(x-x_{0}\right).$$

By analyzing the equations, we can see that the second-order Taylor approximation hardware implementation requires two multipliers, one adder, and two subtractors. The operation of the PAC is described using pseudocode in Algorithm 2.

**Figure 4:**
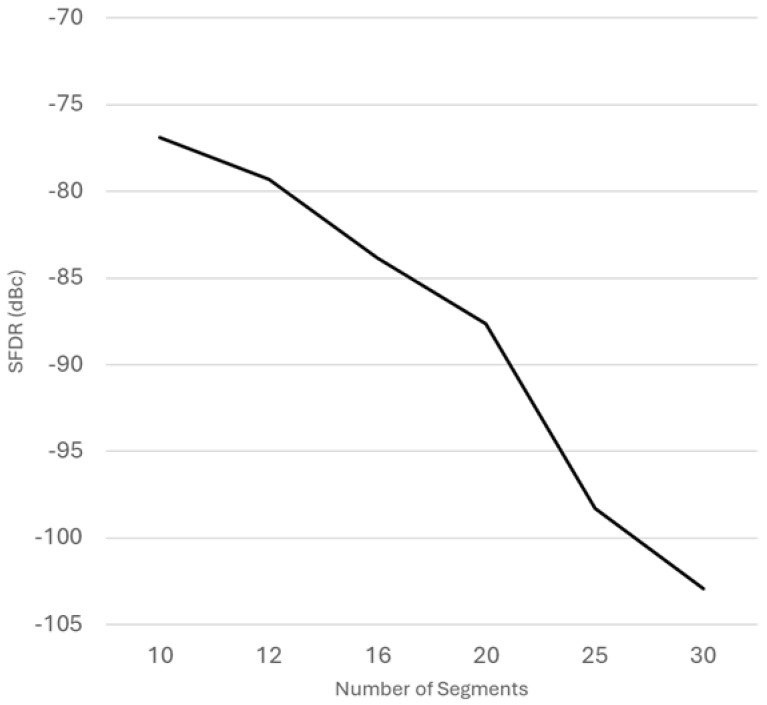
Simulation results showing how adding evaluation points impacts the SFDR. With 30 evaluation points, the second-order Taylor series DDFS can reach SFDR values below −100 dBc.

Next, we need to determine how many segments are required to create accurate amplitude values and provide a high SFDR. For the purpose of the reference design, we will set the SFDR target to below −100 dBc. As is described in Algorithm 2, for each segment, three values need to be stored in the memory: the evaluation point $x_{0}$, and both sine and cosine values at that evaluation point, $sin(x_{0})$ and $cos(x_{0})$. As we are utilizing the eighth-wave symmetry, all the segments are in the approximation interval $\left[0,\frac{\pi }{4}\right]$. Furthermore, since the Taylor series approximation is equally accurate to both directions from $x_{0}$, we will set the evaluation points to be the middle of each segment. A Python model was developed to simulate the SFDR of the second-order Taylor series approximation PAC using a different number of segments. The model was refined to match the finite word length arithmetic of hardware implementation by truncating the computation and using 18 bits for the phase and 16 bits for the amplitude. The Python model was then simulated by varying the number of segments between 10 and 30. Figure 4 depicts the simulation results and how the SFDR improves when the number of segments grows. The memory size and SFDR relationship is summarized in Table 1. Based on the Python simulations, by using 30 segments, the design can reach below −100 dBc SFDR.
**Algorithm 2** The second-order Taylor series-based PAC operation.
    PHASE TO AMPLITUDE CONVERTER    **Input:** Phase word, $P_{\mathrm{word}}$, 18-bit    **Output:** 16-bit sine signal ${\mathrm{Sine}}_{\mathrm{out}}$ and 16-bit cosine signal ${\mathrm{Cosine}}_{\mathrm{out}}$ between $\left[0,\frac{\pi }{4}\right]$             whilesystemondo              $\mathrm{Segment}=\mathrm{DefineSegment}(P_{\mathrm{word}})$              //ReadTaylorcoefficientsfromthememory              $\mathrm{Common}\_\mathrm{multiplicand}=P_{\mathrm{word}}-\mathrm{read}\_\mathrm{memory}\left(\mathrm{Evaluation}\_\mathrm{point}\left[\mathrm{Segment}\right]\right)$              Sine_1st_coeff=read_memory(Sine[Segment])              Cosine_1st_coeff=read_memory(Cosine[Segment])              //Calculatethe2ndtermofthe2ndorderTaylorapproximation              Sine_2nd_coeff=Common_multiplicand·Sine_1st_coeff              Cosine_2nd_coeff=Common_multiplicand·Cosine_1st_coeff              //Calculatethefinaloutput              ${\mathrm{Sine}}_{\mathrm{out}}=\mathrm{Sine}\_1\mathrm{st}\_\mathrm{coeff}+\mathrm{Cosine}\_2\mathrm{nd}\_\mathrm{coeff}$              ${\mathrm{Cosine}}_{\mathrm{out}}=\mathrm{Cosine}\_\mathrm{value}-\mathrm{Sine}\_2\mathrm{nd}\_\mathrm{coeff}$         endwhile             //Functioncalculatesthecorrectsegmentbasedonthephasevalue         FunctionDefineSegment(Phase)              i=1              whilei<Number_of_Segmentsdo                   //Checkifthephaseisthesegmenti                   $\mathrm{if}\;\mathrm{Phase}<{\mathrm{Segment}}_{\mathrm{border}}\left[i\right]\;\mathrm{then}$                         returni                   endif                   //Ifnot,movetothenextsegment                   i=i+1              endwhile


### 3.4. Sine and Cosine Symmetry Logic

The eighth-wave symmetry is implemented in a separate digital logic block. This block receives the control signals from the PA as well as the sine and cosine amplitude values between $\left[0,\frac{\pi }{4}\right]$ from the PAC. The full period of 2π for sine and cosine signals is generated based on the control signals, which define where on the unit circle the amplitude values are being computed. The final 16-bit sine and cosine values are then obtained by correctly rotating, negating, and swapping the PAC output signals.

## 4. Experimental Results and Comparison

**Figure 5:**
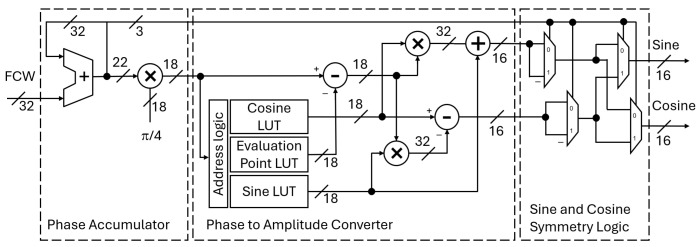
Architecture of the DDFS based on interpolation using the second-order Taylor series approximation. The figure shows the three main components: the phase accumulator, the phase-to-amplitude converter with three LUTs, and sine and cosine symmetry logic. Also, the related signal widths for arithmetic logic are shown.

The second-order Taylor series approximation-based DDFS reference design was chosen to be implemented on an FPGA circuit. The reason to choose FPGA is the possibility for implementing efficiently the multiplication operations required by the design. In an application-specific integrated circuit (ASIC), the multiplication is typically the largest and the slowest arithmetic operation. However, on an FPGA, the multiplication is well optimized. This is because the FPGA manufacturers have hardened the multiply operation in specific digital signal processing (DSP) slices [31]. The target FPGA is AMD XC7A100T, which belongs to AMD’s Artix^TM^ 7-series family. The FPGA provides natively 25 × 18 bit multipliers in its DSP slices. The architecture of the DDFS and the related bit widths have been chosen to take advantage of these DSP slices. An architecture schematic of the DDFS is shown in Figure 5. The PA has one 22 × 18 multiplier for transforming the phase into radians by multiplying the phase register output with $\frac{\pi }{4}$. The resulting product is truncated to an 18-bit phase, which is then provided for the PAC logic.

The size of the memory required for storing the Taylor series coefficients is small, and it does not need to be implemented using actual ROM logic. Instead, the memory can be implemented using digital logic and look-up tables (LUTs). This is beneficial, as the target FPGA, and FPGAs in general, have limited on-chip memory. The implementation has three LUTs that store for each segment the evaluation point and the related sine and cosine values. Since the simulation showed that beyond −100 dBc, an SFDR can be reached with 30 segments, the PAC design needs to store in total 90 values across the three LUTs. Each value is set to 18-bit accuracy, resulting in the total memory of 1620 bits. As described earlier, the traditional DDFS with quadrature output and using a QW memory compression scheme needs to store $2^{q}\cdot m\cdot 2$ values in the ROM. Thus, with the reference design having an 18-bit truncated, QW-compressed phase width and 16-bit amplitude width, we can calculate that the proposed architecture provides a high, 5178:1, memory compression ratio compared to the traditional DDFS with QW-compressed ROM. The high memory compression ratio comes at the expense of some additional PAC arithmetic logic. First, the sine and cosine amplitude computation uses one 18-bit subtractor to compute $x-x_{0}$, which is a shared term between (7) and (8). Then, the resulting difference is multiplied in two separate 18-bit multipliers with one multiplier having sine, $sin(x_{0})$, and the other cosine, $cos(x_{0})$, values coming from the LUT. The final amplitude is calculated in a 32-bit adder for sine and a 32-bit subtractor for cosine.

Finally, the DDFS architecture includes a combinatorial logic circuit that enables the use of the sine and cosine eighth-wave symmetry. The logic is controlled by the three $P_{word}$ MSBs coming from the PA. These bits define in which of the eight sectors on the unit circle the amplitude values should be provided. Based on this, the logic converts the PAC output to one of the three possible outcomes: (1) the signal is unchanged, (2) the signals are swapped with each other (e.g., sin is used for cos and vice versa), and (3) the signal is negated (e.g., sin becomes -sin). The outputs of the logic are the final sine and cosine signals.

### 4.1. Simulation

**Figure 6:**
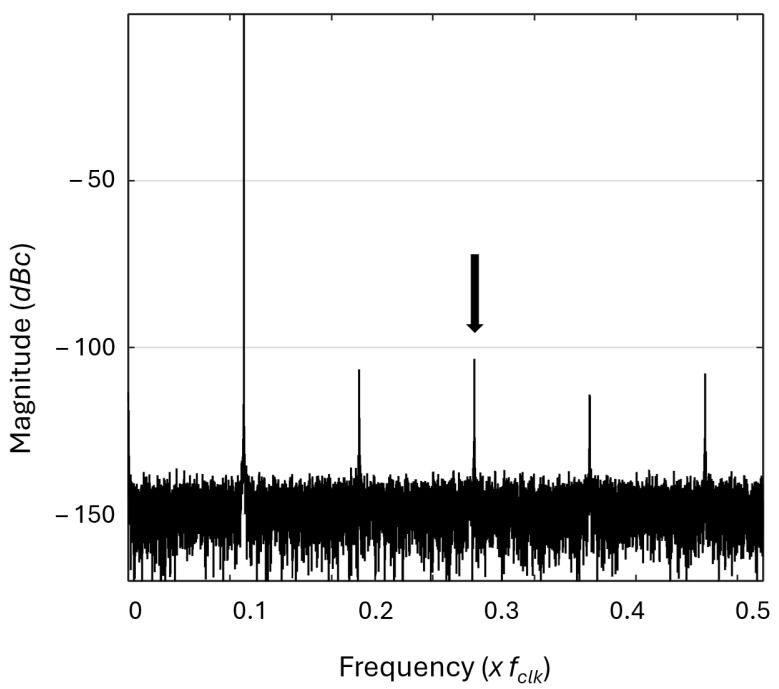
Spurious performance of the sine signal of the second-order Taylor series DDFS. The frequency for the signal is 9.1 MHz ($f_{clk}=100$ MHz). The arrow in the picture points to the largest spur, which is −102.90 dBc.

The architecture from Figure 5 has been implemented using Register Transfer Level (RTL) VHDL. The bit accurate VHDL code was simulated on the logical circuit level in Modelsim, revision 2016.10, Mentor Graphics Corporation (now: Siemens EDA, Wilsonville, OR, USA), functional simulation software using multiple different FCW combinations to validate the DDFS performance. The simulation results were further analyzed using fast Fourier transformation (FFT) on GNU Octave. The implementation results confirmed the Python model results, and the RTL VHDL system was capable of reaching below −100 dBc spurious performance. The FFT spectrum showing −102.90 dBc SFDR for the 9.1 MHz sine signal is depicted in Figure 6. The system clock frequency is 100 MHz. This is limited by the FPGA development board used.

### 4.2. FPGA Implementation

The VHDL code was synthesized using AMD (Advanced Micro Devices, Santa Clara, CA, USA) Vivado Design Suite v.2024.2 (64-bit). The development board used was Digilent (Pullman, WA, USA) Nexys A7, which contains an AMD Artix^TM^ 7 FPGA. To meet the timing requirements and to take full advantage of the DSP optimizations, including lower power, the design was pipelined following AMD’s FPGA design recommendations. Registers were placed both in the inputs and the outputs of the multipliers. These registers were put under synchronous reset, as this enables the synthesis tool to integrate these inside the DSP slices. This integration provides better resource utilization, as the pipelining does not consume registers from the logic slices. To validate this, designs with both synchronous and asynchronous resets were synthesized and tested. The synthesis results showed that using synchronous reset reduced the resource usage by 5% compared to a design using asynchronous reset. In total, the DDFS design used 328 LUTs, 224 flip-flops (FFs), and 3 DSPs—one for each multiplier. It is important to note that here, the LUT does not refer to any memory, but instead, these LUT resources are standard configurable logic elements on AMD FPGAs that are used to implement various logic functions.

**Figure 7:**
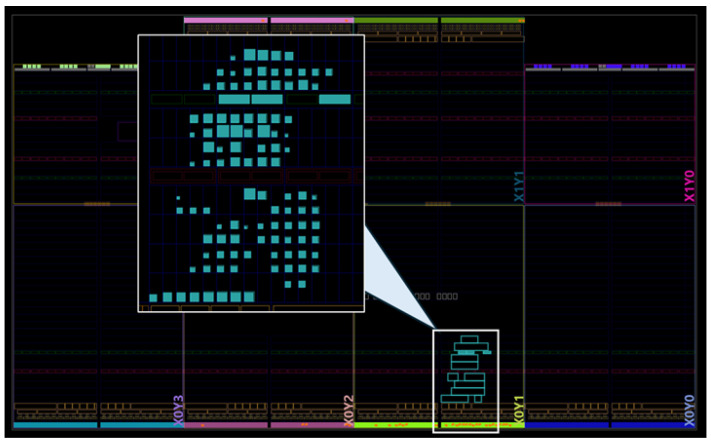
AMD Artix 7 FPGA layout image of the second-order Taylor series-based DDFS design. The blue rectangles in the enlargened image indicate different utilized logic blocks. The three DSP slices are clearly visible in the top part of the design.

The detailed resource distribution between the different architecture components is given in Table 2 and the post place and route FPGA layout image are given in Figure 7. The core dynamic power consumption from clocks, logic, and DSP, excluding signals and I/O, was reported by the Vivado tool to be 10 mW. The power consumption is affected by the environmental conditions, such as airflow and ambient temperature. For the reported power, the ambient temperature was set to 25 °C and the airflow was set to 250 Linear Feet per Minute (LFM). This resulted in the junction temperature of 25.7 °C.

Finally, the implementation results showed that after routing, the design can run at the maximum clock frequency of 130 MHz. The clock frequency requirements are based on the target application. With more aggressive optimization and pipelining, the clock frequency can be increased to meet the application requirements. For example, with synthesis-based power optimizations, the design can reach, without any architecture changes or pipeline stages, 180 MHz clock frequency. However, the high clock frequency leads both to additional hardware and increased power consumption.

### 4.3. Comparison

One of the key results of the proposed architecture is the high memory compression ratio it provides while maintaining good spurious performance. The memory compression ratio has been the focus of continuous research over the past few decades, and the highest previously reported memory compression ratio was, based on the best of the author’s knowledge, 1792:1 [21]. This was reported only very recently, in 2024. The overview of the memory compression research, and comparison of the proposed architecture with earlier work, is presented in Table 3. In the table, we have provided not only FPGA-based implementations but also ASIC implementations and MATLAB (Mathworks, Natick, MA, USA) simulation-based research that have reported high memory compression ratios. From the table, we can see that while the Taylor series has been applied in earlier work, in this proposed architecture, we are using the Taylor series to reach both a higher SFDR and better memory compression ratio than the previous work has reported. Overall, the memory compression ratio presented in the proposed design provides nearly three times improvement over the highest previously reported memory compression ratio. At the same time, the proposed architecture has a high SFDR of −102.9 dBc. When compared with the other designs that have high memory compression ratios, the proposed architecture improves the SFDR by approximately 15 dB.

The proposed second-order Taylor series approximation-based DDFS is compared to other recently published DDFS architectures in Table 4. The previous work designs have been implemented on FPGAs coming from two different manufactures, Intel (Santa Clara, CA, USA, note: Intel has acquired Altera) and AMD (note: AMD has acquired Xilinx). The size comparison between two different FPGA manufactures cannot be made directly, since each manufacturer uses slightly different-sized basic logic elements in their products [33]. For example, AMD uses six-input LUTs, while Intel has two different types of LUT, a four-input LUT and adaptive LUT (ALUT). On AMD, there is also a higher-level architecture block called slice. Taking the data from AMD 7-series FPGAs as a reference, one slice contains four six-input LUTs, eight flip-flops (FF), some multiplexers and carry logic. Thus, the AMD designs can be compared with each other using one of the two separate resource usage metrics, either (1) slices, or (2) LUTs and flip-flops. In order to improve the comparison with previous work, Table 4 lists for the reference design both slice and LUT and FF-based resource utilization metrics.

Looking at the resource utilization in Table 4, let us first focus on the designs that are using AMD/Xilinx manufactured FPGAs, similarly to our reference design. The resource comparison can be made more directly, and we can notice that the proposed method uses significantly fewer resources than the other designs. Also, when comparing the DDFS performance and the output signal purity, we can see that only two designs have reported a higher SFDR at −110.7 dBc and at −114.04 [34,39]. However, a closer observation of these designs reveals that reaching this high SFDR requires 626 interpolation segments in [34] and 100 segments in [39]. The resulting resource utilization is in [34] 12.8 times larger and [39] uses 22% more LUTs than the proposed architecture. Furthermore, the amplitude width in both high SFDR designs is four bits longer than in the proposed design, which reduces the error and improves the accuracy of the output signal. The work in [34] also presents a second, less complex architecture using the same design method. That second design has 141 interpolation segments, 16-bit amplitude resolution, −91.7dBc SFDR, and a resource utilization of 301 slices. Similarly, ref. [39] presents a version using 16-bit output and 31 interpolation segments, making it more comparable with the proposed design. This version consumes 28% less LUTs but 33% more DSPs cells and reaches an SFDR of −91.41 dBc. Since DSP resources are more scarce than LUTs on FPGAs, it can be concluded that the proposed implementation is more efficient than [39]. Taking these designs into account and looking at the data in Table 4, we can conclude that the proposed architecture achieves a high SFDR with low resource utilization when compared with [34] and other designs implemented on AMD FPGAs.

When comparing the proposed design with the Intel and Lattice-based designs, we can observe that the proposed design uses significantly fewer resources for arithmetic and logic than [21,38]. Since the difference in LUT resource utilization is large, we can conclude that the proposed design is more hardware efficient than [21,38]. However, the comparison with [20] is more difficult, as the resource utilization with both approaches is similar. However, by taking into account the data from Table 3, we can notice that the proposed design provides over six times better memory compression ratio than [20], and we can conclude that the proposed design is more efficient.

Finally, in terms of the system clock frequency, the proposed design has average clock frequency when compared with previous work. As was discussed earlier, if the end application requires higher clock frequency, this can be achieved either by synthesis optimization or by adding pipeline stages. Both of these approaches come at the cost of some additional hardware.

## 5. Conclusions

In this paper, we propose a DDFS architecture with a high memory compression ratio and good spurious performance. We start by giving an overview of the various ROM compression methods and present how the previous work has applied the Taylor series approximation. One of the most common ways to reduce the memory size is to leverage the sine and cosine symmetry properties, and we are using the same techniques to optimize the performance of the proposed design. In the proposed DDFS, the amplitude computation is based on interpolation using the second-order Taylor series approximation, and we present how the approximation accuracy can be improved by dividing the amplitude computation into multiple segments.

The proposed method was implemented on an FPGA circuit, and both implementation and simulation results were presented. Based on the results, we concluded that the proposed architecture provides three improvements over the previous work: (1) a high memory compression ratio of 5178:1, (2) good spurious performance with the SFDR at −102.9 dBc, and (3) low FPGA resource utilization, as the design used only 328 LUTs and 224 FFs in 107 slices.

While the proposed architecture already shows great performance over other DDFS approaches, depending on the final application, the DDFS implementation can be further optimized. For example, when SFDR requirements are more relaxed, the bit width of the arithmetic operations can be reduced. This simplifies the logic and enables better implementation, for example, on an ASIC. Also, the memory size can be further reduced by optimizing the individual stored values. As an example, the bit sequence “0111” is replicated 93 times in the LUT values. With some combinatorial logic, this sequence would need to be stored only once, and 368 bits of memory could be saved. This paper shows that the second-order Taylor series approximation-based interpolation is a very viable approach for building DDFSs for various kinds of applications.

## Figures and Tables

**Table 1:** Memory size and SFDR value based on the number of segments.

Segments	Memory Size (Bits)	Memory Compression Ratio ^1^	SFDR (dBc)
10	540	15,534:1	−76.87
12	648	12,945:1	−79.29
16	864	9709:1	−83.84
20	1080	7767:1	−87.63
25	1350	6213:1	−98.27
30	1620	5178:1	−102.90

^1^ The memory compression ratio is calculated by comparing the memory the size with an equivalent QW-compressed DDFS having 18-bit phase and 16-bit quadrature output (i.e., $2^{18}\cdot 2\cdot 16$).

**Table 2:** AMD Artix^TM^ 7 FPGA resource usage by the different Taylor DDFS architecture components.

Design Block	Phase Accumulator	Phase-to-Amplitude Converter	Sine/Cosine Symmetry Logic	Total ^1^
LUTs	196	84	49	328
Flip-Flops	35	116	22	224
Slices	61	37	17	107
DSPs	1	2	–	3
Cells	269	256	46	571
Nets	358	541	109	1008

^1^ Total value is a separate report and not added from columns. It may differ from column values, because due to resource sharing or timing requirements, the synthesis tool may distribute the logic into multiple slices.

**Table 3:** Memory compression ratio development.

Design Approach	Year	Technology/Method	SFDR (dBc)	Memory Compression Ratio
Refs. [23,27], Trigonometric identities	1984	3.5 μm CMOS/SOS	−86.91	59:1
Ref. [27], Taylor series approximation with three terms	1997	MATLAB Simulation	−97.04	64:1
Ref. [29], Taylor series interpolation	2000	0.8 μm CMOS	−77	315:1
Ref. [32], Trigonometric approximation with linear equations	2005	0.22 μm CMOS	−96.67	565:1
Ref. [20], Parabolic interpolation	2012	FPGA	−85	843:1
Ref. [18], Linear interpolation	2014	MATLAB Simulink	−84	1103:1
Ref. [21], Hermite interpolation	2024	FPGA	−88.134	1792:1
Proposed method, Taylor series interpolation	2025	FPGA	−102.9	5178:1

**Table 4:** Comparison of the proposed architecture to other recent state-of-the-art approaches.

Design & Year	Method	FPGA ^1^	Slices	LUTs	Flip-Flops	Arith. Blocks	Clock Freq.	SFDR	Ampl. Bits
Rust et al., 2012 [34]	Linear Interpolation	Xilinx Virtex	1252		36		322 MHz	−110.7	20
Jeng et al., 2014 [20]	Parabolic Interpolation	Altera Stratix II		71 (ALUT)		3 DSPs	60 MHz	−84.71	14
Zhang et al., 2015 [35]	CORDIC	Xilinx Virtex-6	1167	1584	1603		303 MHz	−91.67	16
Annafianto ^2^ et al., 2020 [11]	CORDIC	Xilinx Virtex-6		498	206	2 multipliers, 2 adders	211 MHz	−72.2	16
Mohanty et al., 2022 [36]	CORDIC	Xilinx Spartan 6	1031	1189	992		158 MHz	N/A	16
Changela et al., 2022 [37]	CORDIC	Xilinx Artix 7		1176			200 MHz	−78	20
Xing et al., 2023 [38]	Recursive Trigonometric	Lattice ICE40		1009	1142	1 multiplier	50 MHz	−58.52	16
Liao et al., 2024 [39]	Linear Interpolation	AMD Zynq UltraScale+		399	66	3 DSPs	244 MHz	−114.04	20
Zhou et al., 2024 [21]	Hermite Interpolation	Altera Cyclone II		1392 LEs		46 multipliers	N/A	−88.13	14
Proposed method, 2025	Taylor Interpolation	AMD Artix 7	107	328	224	3 DSPs	130 MHz	−102.9	16

^1^ Using the vendor name listed in the original publication. Xilinx has been acquired by AMD. Altera has been acquired by Intel. ^2^ The design has only PAC implemented. Resource use data does not include PA logic.

## Data Availability

The data presented in this study are available on request from the corresponding author due to privacy reasons.
